# High nevus counts confer a favorable prognosis in melanoma patients

**DOI:** 10.1002/ijc.29525

**Published:** 2015-04-11

**Authors:** Simone Ribero, John R. Davies, Celia Requena, Cristina Carrera, Daniel Glass, Ramon Rull, Sergi Vidal‐Sicart, Antonio Vilalta, Lucia Alos, Virtudes Soriano, Pietro Quaglino, Victor Traves, Julia A. Newton‐Bishop, Eduardo Nagore, Josep Malvehy, Susana Puig, Veronique Bataille

**Affiliations:** ^1^ Department of Twin Research & Genetic EpidemiologyKing's College LondonLondonUnited Kingdom; ^2^ Department of Medical Sciences, Section of DermatologyUniversity of TurinTurinItaly; ^3^ Department of DermatologyLondon North West Healthcare NHS Trust Northwick Park HospitalLondonUnited Kingdom; ^4^ Imperial College LondonLondonUnited Kingdom; ^5^ Section of Epidemiology and BiostatisticsLICAP, University of LeedsLeedsUnited Kingdom; ^6^ Department of DermatologyInstituto Valenciano De OncologıaValenciaSpain; ^7^ Department of DermatologyMelanoma Unit, Hospital Clinic & IDIBAPS, University of BarcelonaBarcelonaSpain; ^8^ Department of SurgeryMelanoma UnitHospital Clinic & IDIBAPS, University of BarcelonaBarcelonaSpain; ^9^ Department of Nuclear Medicine ServiceMelanoma Unit, Hospital Clinic & IDIBAPS, University of BarcelonaBarcelonaSpain; ^10^ Department of Pathology ServiceMelanoma Unit, Hospital Clinic & IDIBAPS, University of BarcelonaBarcelonaSpain; ^11^ Department of OncologyInstituto Valenciano De OncologıaValenciaSpain; ^12^ Department of PathologyInstituto Valenciano De OncologıaValenciaSpain; ^13^ Instituto de Salud Carlos IIICIBER on Rare DiseasesBarcelonaSpain; ^14^ Department of DermatologyWest Herts NHS TrustHertfordshireUnited Kingdom

**Keywords:** nevus count, survival, sentinel lymph node, melanoma

## Abstract

A high number of nevi is the most significant phenotypic risk factor for melanoma and is in part genetically determined. The number of nevi decreases from middle age onward but this senescence can be delayed in patients with melanoma. We investigated the effects of nevus number count on sentinel node status and melanoma survival in a large cohort of melanoma cases. Out of 2,184 melanoma cases, 684 (31.3%) had a high nevus count (>50). High nevus counts were associated with favorable prognostic factors such as lower Breslow thickness, less ulceration and lower mitotic rate, despite adjustment for age. Nevus count was not predictive of sentinel node status. The crude 5‐ and 10‐year melanoma‐specific survival rate was higher in melanomas cases with a high nevus count compared to those with a low nevus count (91.2 *vs*. 86.4% and 87.2 *vs*. 79%, respectively). The difference in survival remained significant after adjusting for all known melanoma prognostic factors (hazard ratio [HR] = 0.43, confidence interval [CI] = 0.21–0.89). The favorable prognostic value of a high nevus count was also seen within the positive sentinel node subgroup of patients (HR = 0.22, CI = 0.08–0.60). High nevus count is associated with a better melanoma survival, even in the subgroup of patients with positive sentinel lymph node. This suggests a different biological behavior of melanoma tumors in patients with an excess of nevi.

## Abstract 2

**What's new?**

Having a lot of pigmented skin lesions known as naevi or moles is a known risk factor for melanoma, but is it also a bad prognostic marker for melanoma patients? The authors looked at the impact of nevus counts on melanoma survival in more than 2000 patients and found the opposite: high nevus counts were associated with favorable prognostic markers and a higher 5‐ and 10‐year survival rate. These findings point to biological factor associated with high nevus count that may confer improved survival and may be exploited therapeutically in the future.

**Abbreviations**
DSS: disease‐specific survival
SLN+: sentinel lymph node positive
SLN−: sentinel lymph node negative

The mean total body nevus count remains the most powerful predictive phenotypic marker for melanoma risk.^1^, ^2^, ^3^, ^4^, ^5^ Nevi typically involute after the fourth decade of life in Caucasian populations, and is attributed to a process of senescence.^6^ Nevi are therefore much less numerous in the elderly.^2^, ^7^ The rate at which nevi disappear with age varies greatly, with some individuals having a large number of nevi in late middle life and this is associated with an increased risk of melanoma.^8^ This implied delayed senescence of nevi may result from loss of tumor suppressor genes such as *CDKN2A* as increased numbers of nevi occur in the carriers of germline *CDKN2A* mutations and mutation in oncogenes such as *BRAF*.^9^ Other studies have suggested that the telomere unit could also play a role in the delayed nevus senescence observed in patients with melanoma .^10^, ^11^ High nevus count has been reported to be associated with longer white cell telomeres as well as increased melanoma risk.^6^, ^12^, ^13^, ^14^ Inherited polymorphisms in genes determining telomere length are associated with increased melanoma risk.^9^, ^15^, ^16^, ^17^, ^18^, ^19^, ^20^ The aim of our study was to investigate whether nevus numbers are predictive of sentinel lymph node (SLN) status and melanoma survival.

## Material and Methods

For this retrospective study, we analyzed data from two different European Countries namely, Spain and United Kingdom. In Leeds (United Kingdom), case–control study, the melanoma cases predominantly derived from a population ascertained series recruited between 2000 and 2005.^21^ In total, 960 cases were recruited as part of a case–control of melanoma in Yorkshire, UK. The remaining 83 further melanoma cases were recruited from other centers on the basis of presenting with a primary melanoma in sun‐protected sites to increase the numbers of these rare subtypes. In Valencia, prospective melanoma cases, seen between January 2004 and July 2013, were all included in the melanoma database of the dermatology department of the Instituto Valenciano de Oncologia (*N* = 463). The characteristics of this database have been described in detail elsewhere.^22^ For Barcelona, melanoma cases submitted to SLN biopsy were collected from the Melanoma Unit at the Hospital Clinic of Barcelona between October 1996 and 2013 and were recruited from Catalonia as well as further a field as the melanoma clinic at the Hospital Clinic Barcelona is a secondary and tertiary referral center (*N* = 678). The Barcelona cohort has been previously described in several studies.^23^, ^24^, ^25^

*In situ* melanoma, noncutaneous melanomas, patients with unknown primaries or without follow‐up were excluded. For patients with multiple melanomas, the most invasive melanoma tumor was included as it is the one that drives the prognosis as reported earlier.^26^ Ulceration was defined according to the AJCC criteria as the absence of intact epidermis over a portion of the primary tumor. The nevus counts were performed by dermatologists (for Valencia and Barcelona, Spain) or trained research nurses (for Leeds, United Kingdom).

All nevus count were performed at the time of the diagnosis of melanoma. The nevus counts in Barcelona were total body nevus counts with linear data. For United Kingdom, the nevus count protocol was also total body nevus counts divided in 17 body parts. The total body nevus counts in the Valencia cohort were using the cutoff values of 25, 50 and 100. Therefore, the data were not linear. To standardize the nevus counts for our study, a cutoff of 50 nevi was used to create two groups of high and low nevus counts (>50 nevi larger than 2 mm in diameter *vs*. ≤50). SLN biopsy was performed according to the guidelines for each institution and the lymph nodes examined according to the standardized protocol of European Organisation for Research and Treatment of Cancer (EORTC). Ethics committee approval was granted for each institution: the ethical committee of the Hospital Clinic of Barcelona, the Leeds University ethics committee application MREC 01/3/057 and for Valencia, the ethics committee approval: Protocol # 2009‐042.

## Statistical Analyses

Pearson's *χ* ^2^ *d* and Student's *t*‐tests were used to compare the categorical and continuous variables, respectively. For variables not normally distributed (*e.g*., Breslow thickness), the values were log transformed for parametric testing. Logistic regression was used to analyze the association between the prognostic factors and the two nevus categories using a cutoff of 50 nevi as well as the association between prognostic factors and SLN status as a binary valuable. Disease‐specific survival (DSS) was calculated from the surgical excision date of the primary melanoma to the date of melanoma death or last checkup. A melanoma‐related death is considered as a death caused by systemic progression of disease in vital sites (brain, visceral involvement) The exact date and cause of death were checked in the Leeds cohort by registry data, annual patient questionnaires, clinical notes and surveys of their primary care physicians; in the Barcelona cohort by digital records of hospitals in Catalonia, shared digital records in Catalonia, national registry data and telephone calls to relatives and in Valencia *via* hospital registries, National Mortality Registry and telephone calls to relatives.

Kaplan–Meier tables were used to calculate the estimated 5‐ and 10‐year DSS. A Cox regression model was used to calculate crude and adjusted hazard rations (HRs) and 95% confidence intervals (CIs) for high nevus count compared to low nevus count, censoring cases that were lost to follow up and cases with nonmelanoma‐related cause of death. The tests based on Schöenfeld residuals and graphical methods using Kaplan–Meier curves showed no evidence that the proportional hazards assumption was violated for nevus count. Models were adjusted for the “country source” to take in to consideration the risk of possible bias in collecting data from three centers. Other variables for adjustments were sex, age, mitoses, Breslow thickness, ulceration, site of primary and SLN status (positive *vs*. negative). Akaike information criterion was used for the selection of model. All statistical tests were two sided. The *p*‐values of <0.05 were considered significant. All data available for the descriptive statistics were used but in the Cox multivariable model the sample size drops to *N* = 724 because of missing data. Information on SLN status had to be available to be included in the Cox model were available. Statistical analyses were performed using Stata/SE12.0 Statistical Software (STATA, College Station, TX).

## Results

### Study population

A cohort of 2,184 melanoma cases from three European studies was analyzed. From Leeds (United Kingdom) 1,043 (47.76%) melanoma cases recruited to a case–control study were included for which detailed nevus count data were available: 960 of these were population ascertained cases recruited from the Yorkshire region (United Kingdm) and were diagnosed from 2000 to 2006 and the rest were melanoma cases recruited for a study of melanoma on nonsun‐exposed sites.^21^ In Spain, 1,141 (52.24%) melanoma cases from wide catchment areas who had SLN biopsy were recruited from two University Hospitals in Barcelona and Valencia from 1996 to 2013.

### Clinical data

Of the 2,184 melanoma cases, 1,180 (54%) were females. The median age at diagnosis was 53 years (range, 20–82). The site of primary melanoma, when available (missing, 150), was head and neck in 252 (12.4%), trunk in 828 (40.7%) and limbs in 954 (46.9%). Overall mean Breslow thickness was 2.27 mm (SD, ±2.21; median, 1.5 mm). The mean thickness of United Kingdom cases was 2.08 ± 0.05 mm (median, 1.3) compared to 2.52 ± 0.06 mm (median, 1.7) in Spain (*p* < 0.001). Ulceration of primary melanoma was present in 495 of lesions (30.6%, 566 data missing). Ulcerated melanomas were significantly thicker than not ulcerated (3.91 ± 0.14 *vs*. 1.76 ± 0.05 mm, *p* = 0.001) and were more common in older people (22.2% in <50 years old *vs*. 33.4% in ≥50 years old, *p* < 0.001) (Table 1).

**Table 1:** Association between nevus number (high *vs*. low) and melanoma characteristics (*p*‐values based on *χ* ^2^ test, Wilcoxon–Mann–Whitney and *t*‐test)

		**Low nevus count**	**High nevus count**	**Total**	***p*‐Value**
Countries	United Kingdom	627 (60.1%)	416 (39.9%)	1,041	<0.001^a^
	Spain	873 (76.5%)	268 (23.5%)	1,141	
Sex	Female	814 (69.3%)	361 (30.7%)	1,175	0.566^a^
	Male	682 (68.1%)	319 (31.9%)	1,001	
Age (years)	Median	57 (20–84)	48.3 (20–76)		<0.001^b^
Age categorical (years)`	<50	541 (59.4%)	369 (40.5%)	910	<0.001^a^
	≥50	937 (75%)	313 (25%)	1,250	
Ulceration	No	779 (69.4%)	344 (30.6%)	1,123	0.001^a^
	Yes	382 (77.2%)	113 (22.8%)	495	
Breslow thickness (mm)		2.45 ± 0.06	1.86 ± 0.07		<0.001^c^
Breslow thickness grouped using AJCC	T1	364 (62.7%)	216 (37.2%)	580	<0.00^a^
	T2	531 (65.1%)	284 (34.8%)	815	
	T3	344 (75.1%)	114 (24.9%)	458	
	T4	227 (79.6%)	58 (20.3%)	285	
Mitotic rate	<5	784 (68.5%)	361 (31.5%)	1,145	0.05^a^
	≥5	291 (76.7%)	104 (26.3%)	395	
Site of primary	Head and neck	196 (77.8%)	56 (22.2%)	252	<0.001^a^
	Trunk	528 (63.7%)	300 (36.2%)	828	
	Limbs	660 (69.2%)	294 (30.8%)	954	
SLN performed	No	531 (59.3%)	364 (40.7%)	895	<0.001^a^
	Yes	969 (75.2%)	320 (24.8%)	1,289	
SLN status	Negative	745 (74.9%)	249 (25.0%)	994	0.732^a^
	Positive	224 (75.9%)	71 (24.0%)	295	

a *p*‐Value calculated using Pearson's *χ* ^2^ *d* test.

b *p*‐Value calculated with Wilcoxon–Mann–Whitney test.

c *p*‐Value calculated using *t*‐test.

High mitotic rate (≥5 mitoses) (when available, 644 missing) was reported in 395 patients (25.6%). Higher mitotic rate was associated with greater Breslow thickness (mean, 1.80 ± 0.05 mm for <5 mitoses *vs*. 4.18 ± 0.17 mm for ≥5 mitoses).

SLN was performed at staging in 1,289 patients (59%) with 1,141 (88.5%) from Spain, whereas the remaining 148 patients (11.5%) were from United Kingdom. In total, 295 patients (22.9%) had a positive SNB status. Sentinel node biopsy was not performed in 41% of the whole melanoma cohort. These patients mainly came from United Kingdom where sentinel node biopsy is less frequently performed compared to Spain or they did not meet the inclusion criteria or declined. There was a nonsignificant protective role of high mole count on SLN status (*p* = 0.74). SLN status was not associated with age or sex in our study. However, SLN status was significantly associated with thicker tumors, ulceration and mitotic rate (Table 2).

**Table 2:** Multivariable logistic regression looking at all melanoma prognostic factors according to SLN status: positive *versus* negative (*N* = 724)

**SLN status**		**OR**	***p*‐Value**	**95% CI**
Nevus count	>50	1.20	0.43	0.76–1.88
Age	Linear	1.00	0.81	0.99–1.01
Breslow thickness (mm)	Linear	1.11	0.002	1.04–1.19
Sex	(Male *vs*. female)	1.13	0.524	0.78–1.64
Site of primary	Head and neck	1		
	Trunk	1.79	0.07	0.95–3.37
	Limbs	1.52	0.21	0.79–2.91
The presence of ulceration	(Yes *vs*. no)	1.82	0.003	1.23–2.70
Tumor mitotic rate	(≥5 *vs*. <5)	1.55	0.03	1.03–2.33
Countries	United Kingdom	1		
	Spain	0.81	0.45	0.47–1.40

### Correlations of melanoma prognostic factors with nevus counts

In all, 684 (31.3%) melanoma cases had high nevus counts (defined as [mt]50 nevi). The results are summarized in Table 1. Age was inversely and highly correlated with high nevus counts as expected (odds ratio [OR], 0.96; *p* < 0.001). Male sex was associated with high nevus counts when age was adjusted (OR = 1.22, *p* = 0.04). Breslow thickness was inversely correlated with high nevus counts (OR = 0.89, *p* < 0.001) as well as ulceration (OR = 0.74, *p* = 0.02) when adjusted for age. Mitotic rate was not associated with high nevus counts when adjusted for age (OR = 0.89, *p* = 0.39). Trunk melanoma was the site most commonly associated with high nevus counts in both sexes (females OR =1 .94, *p* = 0.02; males OR = 1.71 *p* = 0.01).

### Survival and nevus counts

**Figure 1:**
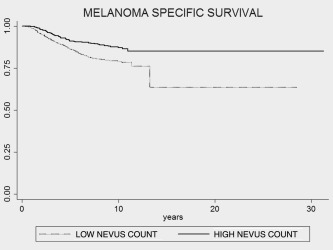
Kaplan–Meier DSS estimates for nevus counts stratified into high nevus counts (>50 mol) and low nevus counts (≤50 mol) in three cohorts from Leeds, Valencia and Barcelona. A significant difference in survival was observed between the high nevus count and the low nevus count group (log‐rank test; *p* < 0.001).

Of the total of 2,184 patients, 283 (12.9%) died during the followup. Median followup was 6.56 years (range, 0.21–12.13). Univariable analyses showed that the known prognostic factors of age, Breslow thickness, sex, site of the primary, microscopic ulceration, mitotic rate and SLN status affected the survival (Table 3). The patients with trunk and limb melanoma had a better prognosis compared to head and neck tumors. The Kaplan–Meier estimates for DSS showed a significantly different survival between those with high *versus* low nevus counts *versus* those with fewer nevi: DSS of 91.16 *versus* 86.4% at 5 years (*p* < 0.001) and 87.18 *versus* 79% at 10 years (*p* < 0.0001) (Fig. 1). The HR for melanoma death in univariable analyses for high nevus count compared to low nevus count in 2,133 patients was 0.47 (CI = 0.24–0.93, *p* = 0.02). Univariate analyses showed that the known prognostic factors of age, Breslow thickness, sex, site of the primary, microscopic ulceration, mitotic rate and SLN status affected the survival (Table 3). In the univariate analyses, nevus counts also improved the survival.

**Table 3:** Univariable and multivariable cox analyses on DSS (*n* = 724)

		Univariable			Multivariable		
		**HR**	***p*‐Value**	**95% CI**	**HR**	***p*‐Value**	**95% CI**
Nevus count	>50	0.47	0.03	0.24–0.93	0.43	0.02	0.21–0.89
Age	Linear	1.03	<0.001	1.02–1.05	1.02	0.02	1.00–1.04
Breslow thickness (mm)		1.15	<0.001	1.10–1.19	1.07	0.02	1.01–1.14
Sex	(Male *vs*. female)	1.76	0.02	1.10–2.81	1.60	0.07	0.96–2.65
Site of primary	Head and neck	1			1		
	Trunk	0.31	<0.001	0.16–0.56	0.23	<0.001	0.12–0.44
	Limbs	0.45	0.01	0.25–0.81	0.46	0.01	0.25–0.84
The presence of ulceration	(Yes *vs*. no)	2.90	<0.001	1.85–4.53	1.30	0.29	0.79–2.14
Tumor mitotic rate	(≥5 *vs*. <5)	4.14	0.000	2.62–6.53	3.00	0.000	1.81–4.97
SLN status	Positive *vs*. negative	4.41	<0.001	3.38–5.75	3.72	<0.001	2.29–6.04
Countries	United Kingdom	1			1		
	Spain	1.20	0.53	0.67–2.14	1.10	0.76	0.59–2.04

This difference in survival according to nevus counts was maintained in multivariable analyses after the adjustment for age, sex, Breslow thickness, ulceration, mitotic rate, site of primary and country source (adjusted HR = 0.43; CI = 0.21–0.89 and *p* = 0.02) (Table 3). Older age, increased Breslow thickness, high mitotic count, site of primary on trunk as well as positive SLN maintained an unfavorable prognostic association for DSS in multivariable analyses (Table 3).

**Figure 2:**
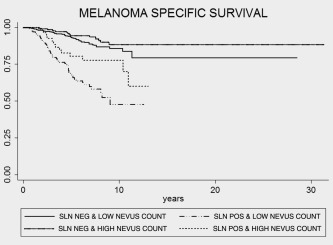
Kaplan–Meier survival estimates for nevus count and SLN status (log‐rank test; *p* < 0.001).

SLN positivity was slightly higher in cases with an excess of nevi but once adjusted for all other melanoma prognostic factors, this did not reach statistical significance. However, SLN status was significantly associated with Breslow thickness, ulceration and mitotic rate as reported before (Table 2). Patients with higher nevus counts seem to receive more frequently SLN biopsy. This could, in part, be explained by age as better prognostic factors with thinner tumors are more common in younger individuals. In fact, the association between being offered a SLN biopsy and high nevus count appeared to be mainly owing to the fact that United Kingdom had the highest percentage of individuals in the high nevus count category and the lowest rates of sentinel node biopsy which affected this association. However, the rate of SNB positivity was similar across the three cohorts. High nevus counts maintained a favorable prognostic role in the positive SLN subgroup (*n* = 174) with an HR of 0.22 (CI = 0.08–0.60) once adjusted for all prognostic factors (Fig. 2 and Table 4).

**Table 4:** Multivariable Cox analyses on DSS in SLN‐positive group only (*n* = 174)

		**HR**	***p*‐Value**	**95% CI**
Nevus count	>50	0.22	0.003	0.08–0.60
Age		1.01	0.44	0.99–1.03
Breslow thickness (mm)		1.09	0.04	1.00–1.19
Sex	(Male *vs*. female)	1.54	0.231	0.76–3.17
Site of primary	Head and neck	1		
	Trunk	0.14	<0.001	0.05–0.39
	Limbs	0.48	0.106	0.19–1.17
The presence of ulceration	(Yes *vs*. no)	0.75	0.428	0.37–1.51
Tumor mitotic rate	(≥5 *vs*. <5)	3.92	<0.001	1.84–8.35
Countries	United Kingdom	1		
	Spain	0.78	0.571	0.32–1.86

## Discussion

Our study shows, for the first time, that having high number of melanocytic nevi at melanoma diagnosis is an independent predictor of better DSS and this protective effect was also observed in patients who have positive sentinel nodes. Melanoma cases with a high nevus count showed a reduction of 57% in melanoma‐specific mortality (HR = 0.43, CI = 0.21–0.89) compared to those with low nevus count after all adjustments. High nevus count is a strong predictive risk factor for cutaneous melanoma.^2^, ^3^, ^4^, ^27^ Nevi involute with age, a process which is likely to be, in part, genetically driven.^28^, ^29^ The improved survival in melanoma cases with high nevus counts raises the possibility that the genetic determinants of nevi number may be associated with biological differences in melanoma tumors.

A potential weakness of our study is that the analyses were carried out in three different melanoma cohorts from the United Kingdom and Spain. The fact that the three centers had slightly different inclusion criteria for entry into the respective melanoma cohort with only the United Kingdom study being population‐based, whereas Valencia and Barcelona were retrospective studies from large melanoma secondary and tertiary referral centers were addressed at least, in part, by adjusting for all melanoma prognostic factors. The distribution of these prognostic factors proved to be very similar between the three centers with slightly thicker tumors in Spain, owing to the inclusion criteria, as patients being offered only SLN biopsy were included in the Spanish melanoma cohorts. The differences regarding the recommendations for SLN biopsy in the three different centers were also a weakness with United Kingdom having a very low rate of SLN biopsy. However, we adjusted for the source of the cohort. Furthermore, as there is no evidence that having SLN biopsy affects survival, the differences in SLNB practices between UK and Spain are unlikely to have been an issue for the survival analyses.^30^ There is no reason to speculate that nevus counts may be altering the decision of a clinician to offer sentinel node biopsy or the patient to accept the procedure apart from age.

Nevus cell senescence may occur, at least in part, because of telomere attrition as the telomere unit has been linked to both high nevus number and melanoma risk.^6^, ^16^, ^18^, ^31^, ^32^ Telomere attrition is reported to trigger the induction of tumor suppressor proteins including p16 (INK4a) and many other cell‐cycle genes.^10^, ^11^ Age did not impact on SLN status in our study in contrast with the results of Balch *et al*.^33^ but we did not have so many cases in the extreme of ages as seen in the Balch series (such as under 20 years or older than 80 years). Breslow thickness, truncal tumors and mitotic rate maintained unfavorable prognostic roles in Stage III as reported earlier.^34^, ^35^, ^36^, ^37^

Higher number of nevi was associated with more favorable prognostic factors in terms of tumor characteristics such as thinner melanoma, less ulceration and fewer mitoses. Higher numbers of nevi were more frequent in participants with trunk melanoma, confirming data from a previous large‐pooled analysis of melanoma case–control studies.^27^ However, the protective effect of higher nevus counts on survival was independent of these well‐established melanoma prognostic factors as the association remained after all adjustments. Other factors may possibly affect the number of nevi and may need to be further investigated. For example, a history of eczema has been associated with lower nevus counts, possibly because of increased immune activation in the skin.^38^ However, all melanoma survival analyses reported in the literature have, so far, not collected any data on dermatosis and hence this cannot be checked for our study. The so‐called divergent aetiology hypothesis which has been explored by many groups^39^, ^40^, ^41^ is that melanoma may arise through at least two different phenotypic pathways: one in younger people, associated with melanocyte proliferation and thus high nevus count, more likely to occur on the trunk, the other with a more frequent localization on the head and neck in elderly people without nevi but more sun damage. The improved survival in patients with high nevus counts supports the view that the biology of different types of melanoma in terms of cutaneous phenotypic risk factors may impact on the survival.

The explanation for a protective effect of an excess of nevi (and implied longer telomeres) on melanoma survival is not clear but may reflect biological differences driven by genes involved in nevogenesis or inherited variations in host responses to the tumor which may be greater in the presence of many nevi. We speculate that as high nevus count is associated with longer white cell telomeres, this might be a marker of delayed senescence of cells of many other lineages with a possible influence on the immune system as well.^6^, ^42^ Hence, one hypothesis is that longer telomeres in immune cells from patients with many nevi might, therefore, be beneficial. The immune system plays a fundamental role in melanoma progression. Older age at diagnosis is an independent unfavorable prognostic factor even after adjusting for all other melanoma prognostic factors.^43^, ^44^ The age effect on survival might be owing to the differences in the aggressiveness of the tumor and/or altered host response to the disease as well as changes in lymphatic flow, comorbidities, or a combination of these factors. The immune system is susceptible to age‐related changes especially in the CD4 compartment with an expansion of CD4 Tregs with age.^45^ Telomere length and telomerase activity also have effects on the immune system with the expansion of CD4+ and CD25+ regulatory T cells with shorter telomeres.^46^ A second hypothesis is that the beneficial effect of nevus number on survival may reflect a modifying effect on tumor cell telomeres. A direct effect of white cell telomere length on melanoma survival has not been clearly proven. Although it has been suggested that shorter white cell telomere length could be associated with a reduced melanoma survival which supports the role of telomeres in melanoma survival and in the same direction as our data.^47^ The effect of telomere length has already been reported in colon cancer as accelerated telomere erosion is associated with a worse survival,^48^ a phenomenon reported to be associated with a decline of immune functions believed to be caused by a shortening of telomeres.^49^ More recent data suggest that somatic alterations in the TERT gene are associated with a worse melanoma prognosis, and hence these results may appear to conflict with our results. It is, however, unknown whether these TERT mutations present in the tumor lead to longer white cell telomere length in these studies.^32^, ^50^, ^51^

High nevus counts, while increasing melanoma risk, appear to have a favorable independent prognostic significance in patients with melanoma implying biological differences in melanoma tumors explained by genetic or environmental factors linked to high nevus counts which could be mediated by telomere biology and/or immune responses. This has implications for melanoma biology and clinicians should be encouraged to record nevus counts in patients with melanoma in clinical practice to assess the role of the nevus phenotype in melanoma survival as well as responses to immunologic and gene‐targeted treatments.
